# Phaseolin, a Protein from the Seed of *Phaseolus vulgaris*, Has Antioxidant, Antigenotoxic, and Chemopreventive Properties

**DOI:** 10.3390/nu13061750

**Published:** 2021-05-21

**Authors:** Juan Manuel García-Cordero, Nikte Y. Martínez-Palma, Eduardo Madrigal-Bujaidar, Cristian Jiménez-Martínez, Eduardo Madrigal-Santillán, José A. Morales-González, Rogelio Paniagua-Pérez, Isela Álvarez-González

**Affiliations:** 1 Laboratorio de Genética, Escuela Nacional de Ciencias Biológicas, Instituto Politécnico Nacional, Av. Wilfrido Massieu s/n. Zacatenco. Gustavo A. Madero, Ciudad de Mexico 07738, Mexico; ibq_juan@live.com (J.M.G.-C.); nicky_yol@hotmail.com (N.Y.M.-P.); edumadrigal.bujaidar@gmail.com (E.M.-B.); 2 Laboratorio de Compuestos Bioactivos, Departamento de Ingeniería Bioquímica, Escuela Nacional de Ciencias Biológicas, Instituto Politécnico Nacional, Av. Wilfrido Massieu s/n. Zacatenco. Gustavo A. Madero, Ciudad de Mexico 07738, Mexico; crisjm_99@yahoo.com; 3 Laboratorio de Medicina de la Conservación, Escuela Superior de Medicina, Instituto Politécnico Nacional, Plan de San Luis y Díaz Mirón s/n. Casco de Santo Tomás, Ciudad de Mexico 11340, Mexico; eomsmx@yahoo.com.mx (E.M.-S.); jmorales101@yahoo.com.mx (J.A.M.-G.); 4 Servicio de Bioquímica, Instituto Nacional de Rehabilitación, Av. Mexico-Xochimilco 289, Ciudad de Mexico 14389, Mexico; rogelpp@yahoo.com

**Keywords:** phaseolin, antioxidant, chemoprevention, antigenotoxic

## Abstract

The present report was designed to determine the antioxidant and antigenotoxic effects of phaseolin (isolated from *Phaseolus vulgaris*) against mouse colon and liver damage induced by azoxymethane (AOM) and its colon chemopreventive effect. Eight groups with 12 mice each were utilized for an eight-week experiment: the control group was intragastrically (ig) administered 0.9% saline solution; the positive control group was intraperitoneally (ip) injected with 7.5 mg/kg AOM twice a week (weeks three and four of the experiment); three groups were ig administered each day with phaseolin (40, 200, and 400 mg/kg); and three groups were ig administered phaseolin daily (40, 200, and 400 mg/kg) plus 7.5 mg/kg AOM twice a week in weeks three and four of the experiment. The results showed that phaseolin did not produce oxidative stress, DNA damage, or aberrant crypts; in contrast, 100% inhibition of lipoperoxidation, protein oxidation, and nitrites induction generated by AOM was found in both organs, and DPPH radical capture occurred. The two highest phaseolin doses reduced DNA damage induced by AOM in both organs by more than 90% and reduced the AOM-induced aberrant crypts by 84%. Therefore, our study demonstrated the strong in vivo antioxidant, antigenotoxic, and chemopreventive potential of phaseolin.

## 1. Introduction

Plants have been used as therapeutic agents dating back to ancient civilizations. Presently, it is known that such an effect may be explained by the presence of diverse plant bioactive compounds, a finding that can be related to the approximately 25% of prescribed medications having a vegetal source origin [1,2].

Leguminosae is a family composed of approximately 700 genera and 2000 species that has also contributed therapeutic properties to the treatment of various diseases, such as diabetes, hypercholesterolemia, inflammation, and helminthiasis, or because of its kidney protective properties [3,4]. Beans (*Phaseolus vulgaris*), one of the most consumed seeds of the group, is a plant of Mesoamerican origin with a dispersion center located in central Mexico [5]. The world production of the common bean is approximately 24 million tons, led by the Latin American/Caribbean region and the Sub-Saharan African region with 24% of the production, and a mean per capita per day consumption led by Latin American and the Caribbean, Sub-Saharan Africa, and South Asian regions, with 34, 33, and 33 g, respectively [6].

Beans are recognized as a good source of protein, carbohydrates, fiber, vitamins, minerals, and phytochemicals. In addition to its nutritive value, studies on *P. vulgaris* have also demonstrated beneficial effects regarding cholesterol levels, cardiac disease, obesity, diabetes, and cancer [7].

In addition to specific therapeutic properties, in recent years attention has been focused on the antigenotoxic effect exerted by plants or their constituents. This area of research has been stimulated by the fact that various diseases, as well as the process of ageing, are known to involve DNA damage in their origin or in their development [8,9]. DNA structure and function may be affected in different forms, one of which is excessive endogenous or exogenous oxidative alterations; therefore, the identification and use of antigenotoxic agents with antioxidant properties is one of the strategies that can be applied against DNA damage. Such protective activity on the damage of the genetic material is also relevant to the prevention of cancer, in light of the fact that such disease, among other events, is usually characterized by the presence of different genetic alterations and increased oxidative stress [10].

Various constituents of *P. vulgaris* have been studied regarding their antigenotoxic potential. Phenols were shown to have this effect in strains of *Salmonella typhimurium* damaged with aflatoxin B1 or benzo (a) pyrene, as well as by using micronucleus and comet assays in the cells of mice treated with cyclophosphamide [11,12,13]. Moreover, a correlation between the antimutagenic and antioxidant effects has also been reported for phenolic compounds obtained from a methanolic extract of the bean seed coat in *Salmonella* exposed aflatoxin B1 and evaluated with beta-carotene and DPPH assays [14]. A number of antioxidant properties have been reported with respect to *P. vulgaris*. For example, it was determined that its methanolic extract, its proanthocyanidin-rich fraction, and its whole bean consumption have significant anti-radical capacity [14,15,16]. In addition, with regard to cancer chemoprevention, epidemiologic studies have suggested that the consumption of beans is associated with a reduction in the rate of breast, prostate, and colon cancers [17,18]. Several parts or specific compounds of *P. vulgaris* are known to experimentally act on various types of cancers; however, no studies about the antigenotoxic and chemopreventive effects of phaseolin have been reported.

Colon cancer in particular is one of the leading causes of death worldwide. The largest fraction of colon cancer cases has been linked to environmental causes rather than heritable genetic changes [19]. Risk factors include environmental and food-borne mutagens, specific intestinal commensals and pathogens, and chronic intestinal inflammation [20]. The pathogenesis of colon cancer involves the sequential and multistep progression of initiated epithelial cells towards a cancerous state with defined precancerous intermediaries. In the process, colon aberrant crypts (CAC) represent the earliest identifiable intermediate pre-cancerous lesions in both laboratory animals and humans [21,22]. On the other hand, epidemiologic data have suggested that this type of cancer may be reduced in populations consuming beans [17,23]. Moreover, this suggestion has been confirmed in experimental assays. For example, Vergara-Castañeda et al. [24] demonstrated that the cooked Bayo Madero variety of *P. vulgaris* and its non-digestible fraction suppressed CAC formation in rats induced with azoxymethane (AOM), and Feregrino-Pérez et al. [25] showed that polysaccharide extract from cooked beans decreased the number of pre-carcinogenic CAC lesions in AOM-treated rats and modified the transcriptional expression of various genes.

The protein content of beans represents approximately 21–24% of their composition, with globulins representing the highest proportion (54–79%) [26]. Such proteins are classified as 7S and 11S according to their sedimentation coefficient. The first mentioned fraction generally corresponds to phaseolin and represents 40–50% of the total seed nitrogen [17]. Phaseolin is a protein containing a neutral sugar, usually mannan, and it is organized into three subunits, each of which possesses different molecular weights, isoelectric points, and glycosylation degrees [27,28]. The nutritive value of phaseolin is limited due to highly resistant in vitro hydrolysis and in vivo digestion (digestibility values ranging from 28 to 36%); however, after thermal treatment, the digestibility can increase considerably (80–90%) [29]. Despite its high abundance in beans, very few biological activities have been reported for this protein; however, Carrasco-Castilla et al. [30] obtained a protein hydrolysate from phaseolin and reported antioxidant activity against a generator of free radicals (ABTS), as well as ferric and copper chelating activity in vitro. Therefore, considering the beneficial biomedical activities of the *P. vulgaris* seed and its various constituents, as well as the relevance of phaseolin and the absence of research on this protein, the aims of the present report were the following: to determine phaseolin’s capacity to inhibit oxidative damage in AOM-treated mouse colon and liver, to examine its antigenotoxic potential in the same AOM-treated mouse organs, and to determine its inhibitory effect on the amount of AOM-induced mouse CAC.

## 2. Material and Methods

### 2.1. Chemicals and Mice

Acetone, HCl, ethyl acetate, formaldehyde, acetic acid, saline solution 0.9%, methanol, and ethanol were acquired from Baker (Phillipsburg, NJ, USA). AOM, dimethyl sulfoxide (DMSO), thiobarbituric acid (TBA), trichloroacetic acid (TCA), 2,4-dinitrophenylhydrazine (DNPH), sodium dodecyl sulfate (SDS), hydroxymethyl aminomethane (TRIS), bromophenol blue, Coomassie brilliant blue R-250, phosphate buffered saline (PBS), guanidine hydrochloride, NaOH, ethidium bromide, NaCl, NaNO_2_, ethylenedinitrilotetraacetic acid (EDTA), polyacrylamide, β-mercaptoethanol, methylene blue, normal melting point agarose, low melting point agarose, lauroyl sarcosinate, Na_2_EDTA, and Triton-X100 were purchased from Sigma Chemicals (St Louis, MO, USA).

To carry out the experiment, we obtained 96 male mice (CD1) from Harlan Laboratories (Mexico City, Mexico) with a mean weight of 23 g. The animals were organized in groups of 12 individuals each and placed in polycarbonate cages at 24 °C, 12-h dark/light cycles, 50% relative humidity, and with free access to water and food (Rodent Lab Chow, 5001, Purina. St Louis, MO, USA). The experiment was approved by the Bioethics Committee of the Hidalgo State Autonomous University (Mexico) and was started after a week of animal stabilization in the Genetics Animal Facility, according to the previously indicated conditions. In addition, we confirm that all methods used in the present research were performed according to the recommended international guidelines and regulations, as reported in the ARRIVE guidelines, published at https://arriveguidelines.org (accessed on 19 August 2019) [31]. The code issued by the Committee of Ethics is as follows: CICUAL-F017-2019.

### 2.2. Isolation of Phaseolin

We initially obtained 2.5 kg of beans (*Phaseolus vulgaris*) cultivated in San Juan del Rio, Querétaro, Mexico, during spring–summer 2018. Initially, the outer head and cotyledons of the seeds were removed prior to grinding and passed through a 0.2-mm mesh. Then, to eliminate phenolic compounds, the obtained flour was dissolved five times in 75% acetone in a proportion of 1:10 *w*/*v* at 4 °C. Each exchange of the solvent was maintained for 30 min. Finally, the excess acetone was eliminated in an extraction chamber at room temperature. To isolate phaseolin, we followed the method proposed by Montoya et al. [32] with some modifications. The flour was suspended in a mix composed of NaCl 0.5 M plus HCl 0.025 M in a proportion of 1:20 with constant agitation for 1 h at 4 °C. After that process, we added distilled water and centrifuged the mixture at 13,500× *g* for 20 min at 4 °C. Then, the precipitated product was washed with distilled water and centrifuged again under the described conditions. This last precipitate was dialyzed with distilled water for 24 h at 4 °C, including an exchange of the water at 12 h to initiate the process. The dialyzed protein was centrifuged at 13,500× *g* and 4 °C for 20 min, and finally, the protein was lyophilized at −47 °C and 0.5 mBar.

#### Phaseolin Identification

Protein characterization was performed using the method described by Laemmli [33], which uses electrophoresis in a polyacrylamide gel, with SDS as the denaturing agent. For this purpose, we used a Mini-Protean 3 Gel Electrophoresis Unit (Bio-Rad, Hercules, CA, USA). The separator gel was 17% polyacrylamide at pH 8.8, and the stacking gel was made of 5% polyacrylamide at pH 6.8. Phaseolin was dissolved in a buffer composed of TRIS-HCl 0.1 M at pH 6.8, 2% SDS, 5% β-mercaptoethanol, and 0.02% bromophenol blue. Phaseolin (15 µL, equivalent to 75 µg of protein/well) was placed into the gels to carry out electrophoresis at 110 V for 90 min. Gels were stained with a solution of 0.125% (*w*/*v*) of Coomassie brilliant blue R-250 made in 7% acetic acid plus 40% methanol (*v*/*v*). Finally, the gels were de-stained by using a solution of 7% acetic acid with 30% ethanol (*v*/*v*). The molecular weight of each band was determined by comparison with a well-known molecular weight standard (All Blue pre-stained protein standard). The protein content from the isolated protein was determined according to the method of Kjeldahl-Gunning 954.01 [34] and was calculated as the percentage of nitrogen × 6.25.

### 2.3. Mouse Experimental Design

Eight groups with 12 mice each were organized as follows for an eight-week experiment: the control group received standard food and water ad libitum and the administration of saline solution 0.9%. All other groups were fed similarly to the control group; however, they differed between each other according to the administration of the tested compounds. A second, positive control group was intraperitoneally (ip) injected with 7.5 mg/kg AOM twice a week during weeks three and four of the experiment; these animals were expected to develop DNA and pre-neoplastic damage (CAC), as well as oxidative alterations. To examine the intrinsic damaging potential of phaseolin, the next three groups were intragastrically (ig) administered each day with the protein at doses of 40, 200, and 400 mg/kg; finally, the last three groups were also ig-administered phaseolin daily at the mentioned doses (40, 200, and 400 mg/kg), but in these groups the mice were also ip treated with 7.5 mg/kg AOM twice a week in weeks three and four of the experiment. Therefore, these three groups were designed to examine the preventive potential of phaseolin against the damage induced by AOM. In all groups administered phaseolin, the compound was given for eight weeks, that is, two weeks before AOM administration. The low dose of the protein was established according to the per capita consumption of beans in Mexico [35], and the medium and high doses corresponded to 5 and 10 times the low dose, respectively. In our study, six mice per group were used for each determination: protein content, lipid peroxidation, protein oxidation, nitrates/nitrites, DPPH assay, comet assay, and CAC. With respect to the registration of body weight, however, we considered twelve mice per group. Besides, in order to have a control positive group for the ex vivo DPPH assay, at the end of the study we orally administered 2571.5 mg/kg of the well-known antioxidant vitamin C to a new group, and 2 h later a retro-orbital blood sample was obtained.

Weight was registered every third day throughout the experiment. At the end, animals were cervically dislocated, and the liver and colon were dissected for study. Six mice from each group were used to obtain information about the protein content, as well as to carry out the comet assay and molecular oxidation assays (lipids, proteins, and nitrites), while the other six animals were used to examine the number and type of CAC.

For the determination of the total protein content and lipid and protein oxidation, a tissue homogenate of both organs (colon and liver) was prepared in PBS (0.01 M at pH 7.4) at a 1:10 (*w*/*v*) ratio. In the case of nitrites, a small portion of both organs was placed in fetal bovine serum plus 15% DMSO (*w*/*v*) and kept at −70 °C until use.

### 2.4. Total Protein Content

For this determination, we followed the method described by Bradford [36]. Initially, 100 µL of the homogenate (colon and liver) was centrifuged at 100× *g* for 10 min, and then 10 µL of the supernatant was mixed with 90 µL of deionized water and 2.5 mL of Bradford’s reactive solution with constant agitation. After 5 min, samples were spectrophotometrically read at 595 nm against a blank consisting of 100 µL of deionized water plus 2.5 mL of Bradford’s reactive. The results were interpolated from a standard curve made with bovine serum albumin (from 0.1 to 1.0 mg/mL) and expressed as mg of protein/g of tissue.

### 2.5. Lipid Peroxidation

This determination was made in terms of the amount of thiobarbituric acid reactive species (TBARS) following the method described by Buege and Aust [37] with some modifications. For this purpose, 500 µL of homogenate (colon and liver) was placed in amber plastic tubes with 2 mL of reactive TCA-TBA-HCl (14%-0.375 *w*/*v*-0.25 N). The mix was boiled for 15 min, placed on ice for 10 min and centrifuged at 2000× *g* for 10 min. Then, the supernatant was read at 534 nm against a blank (500 µL of PBS + 2 mL of TCA-TBA-HCl). The concentration of malondialdehyde (MDA) for each sample was calculated by using an extinction coefficient of 1.56 × 10^5^ M^−1^ cm^−1^, and the results were expressed as µmol MDA/mg of protein.

### 2.6. Protein Oxidation

For this determination, we quantified carbonyl groups according to the method described by Levine et al. [38]. Initially, we added 500 µL of DNPH (10 mM made in 2 M HCl) to the tissue homogenate of both organs. The mix was left in the dark at room temperature for one h, and then the generated hydrazones were precipitated with 500 µL of 20% TCA. Each sample was centrifuged three times at 10,000× *g* for 10 min each, and after each centrifugation, the suspension was washed with 1 mL of ethyl acetate-ethanol (1:1). The obtained pellet was resuspended in 1 mL of guanidine hydrochloride 6 M, incubated at 37 °C for 15 min, and centrifuged at 10,000× *g* for 10 min. For each sample, a blank (500 µL of HCl 2 M) was concomitantly processed. The carbonyl content was registered in a range of 350 to 375 nm, and its concentration was calculated by using 22,000 M^−1^ cm^−1^ as the coefficient of molar absorbance. The results were expressed as µmol of CO/mg of protein.

### 2.7. Nitrates/Nitrites

The tissue previously kept at −70 °C was washed with cold PBS (0.01 M at pH 7.4). For each determination, we prepared a homogenate of each organ with PBS (1:4). The mixture was centrifuged at 2000× *g* for 20 min, after which 100 µL of the supernatant was treated with 300 µL of the Griess reactive plus 500 µL of distilled water [39]. The mix was read at 540 nm, and the obtained absorbances were interpolated from a curve type constructed with NaNO_2_ 0.1 M, from 0.1 to 10 µmol. The results were expressed as µmol of nitrite/g of tissue.

### 2.8. Ex Vivo Antioxidant Capacity of Phaseolin

For this measurement, we used the same eight experimental groups of mice described before (mouse experimental design) plus another group administered vitamin C at a dose of 2571.5 mg/kg (equivalent to 2000 times the recommended daily intake RDI = 1.28 mg/kg/day). The determination was made according to the method of Chrzczanowicz et al. [40]. A retro-orbital blood sample was taken from each animal (six mice per group) and centrifuged at 1500× *g* for 10 min at 4 °C. Then, 200 μL of the obtained serum was mixed with 200 μL of acetonitrile 9.5 M, and a new centrifugation was performed at 10,000× *g* for 10 min at 4 °C. Later, 25 μL of the supernatant was mixed with 5 μL of DPPH 0.01 M plus 970 μL of methanol for 30 min. Then, the absorbances were read at 517 nm in a UV-VIS spectrophotometer. The results are expressed as the percentage of antioxidant capacity (% cAOX).

### 2.9. Comet Assay

A small portion of liver was placed in a Petri dish containing 200 µL of PBS to make a fine tissue suspension with the aid of scissors and was kept in Eppendorf vials. In addition, a small portion of colon was transversally sectioned, placed in another Petri dish, and scraped with the aid of a slide to obtain a cell suspension that was also kept in Eppendorf vials.

The assay was based on published guidelines [41] with various modifications. The number of cells for both tissues was adjusted to 10^5^ cells/mL, and the suspension was kept on ice during the procedure. On fully frosted microscope slides, we placed 100 µL of 1% normal melting point agarose and left it to solidify. Then, a second layer formed by 10 µL of cell suspension plus 65 µL of 1% low melting point agarose was added on top of the first layer. After its solidification, a last layer was added (100 µL of 1% low melting point agarose). Slides were then immersed (for 24 h at 4 °C in the dark) in a lysis solution composed of 1% lauroyl sarcosinate, NaCl 2.5 mM, Na_2_EDTA 100 mM, 1% Triton X-100, 10% DMSO, and 10 mM Tris (pH 10). After that, slides were placed in a horizontal electrophoresis chamber adjusted to pH ≥ 13 with buffer (EDTA 1 mM, NaOH 300 mM). After 20 min of DNA denaturation, the electrophoresis of nucleoids was carried out for 30 min at 25 V, 300 mA, and pH ≥ 13. After the indicated process, slides were dehydrated with 50% ethanol and kept at room temperature until nucleoid observation. For this purpose, nucleoids were stained with 0.02% ethidium bromide, observed under an epifluorescence microscope and analyzed by an image program analyzer (Image Pro-Plus version 5, Media Cybernetics. Rockville, MD, USA). The entire length of the comet and the diameter of the head were measured in 100 nucleoids per mouse. The final measurement was calculated as the length to width diameter [42].

### 2.10. Colon Aberrant Crypts

For this determination, we followed the method described by Bird [43] with some modifications. The colon of each mouse was longitudinally opened, extended and fixed with pins to the bottom of a Petri dish containing a layer of solidified paraffin. The tissue was fixed with 10% formaldehyde made in PBS 0.1 M at pH 7.4 for 24 h at room temperature. After this process, the fixative was discharged, the organ was washed in distilled water, placed in another Petri dish with the mucosal side up, and stained with 0.25% methylene blue in PBS for 10 min. The number and types of CAC (simple and multiple) were identified with a Nikon Eclipse 80i microscope (Nikon Co., Tokio, Japan) at 100× magnification.

### 2.11. Statistical Analysis

Statistical analysis for the obtained data in all tests was carried out by means of a one-way ANOVA followed by the Holm-Šídák test using the Sigma Plot software, (version 12.0, Kansas City, MO, USA).

## 3. Results

### 3.1. Obtention of Phaseolin

**Figure 1:**
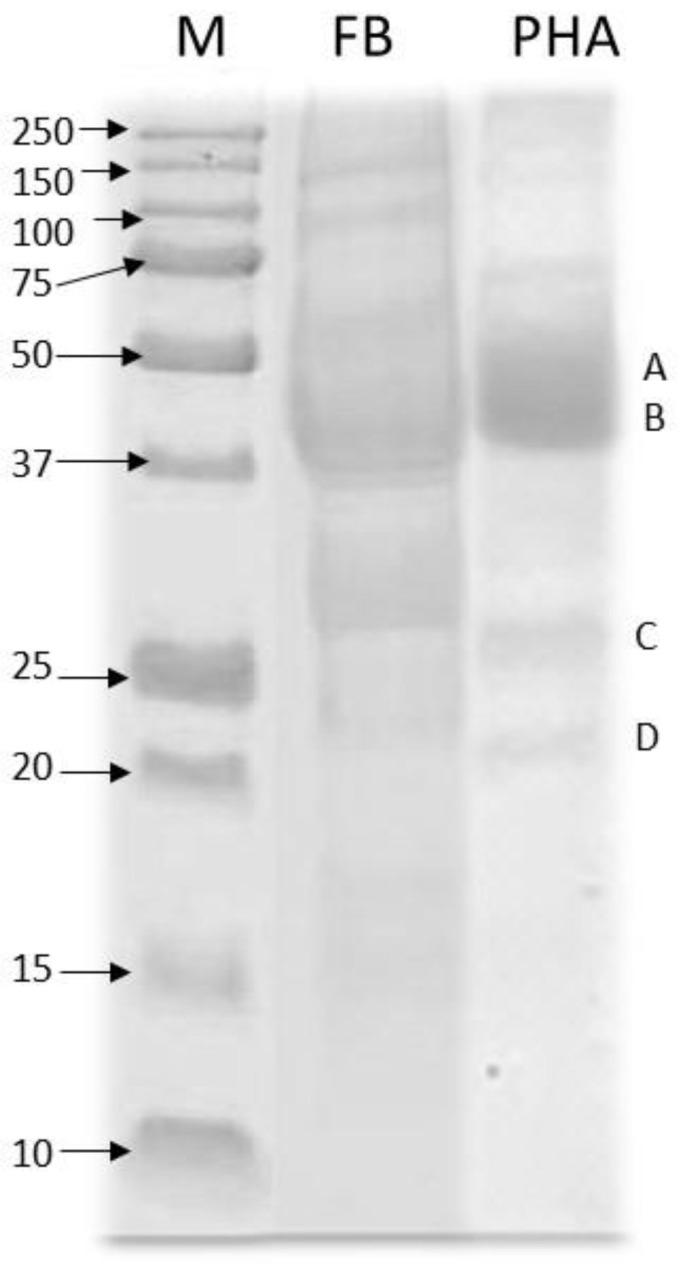
Electrophoretic (SDS–PAGE) pattern of P. vulgaris flour (FB) and phaseolin (PHA). Lane M = MW standards (kDa); Lane FB = bean flour; Lane P = phaseolin extract. Protein bands: (**A**): Phaseolin (43–47 kDa); (**B**): PHA-E, PHA-L (31 kDa); (**C**): Phaseolin b type (fragment, 25 kDa); (**D**): Phaseolin a type (fragment, 21 kDa).

The total protein content in the seeds of *P. vulgaris* was 18.6%, while in the protein isolate this content was substantially increased to 84%. Similar data were previously reported by Clemente et al. [44] in chickpea protein isolates and by Carbonaro [45] in a 7S globulin isolate from common beans. The extract yield of phaseolin was 21%, which is somewhat lower than that obtained in other reports [30,46] and may be related to variations in the protein concentration in different bean varieties and harvests. Figure 1 shows the identification of the proteins present in beans, including phaseolin. Lane 1 (standard) shows proteins with molecular masses between 10–250 kDa, and the lane of phaseolin shows subunits from 45 to 21 kDa with two main bands, at 43–47 kDa (A) and 31 kDa (B). Three bands of low intensity were also observed at 56.78, 24.77, and 19.88 kDa, a similar finding to that reported by Montoya et al. [32], which suggests the purity of the protein.

### 3.2. Mouse Weight Gain

The registration of mouse weights throughout the experiment is shown in Table 1. A mean increase of 6.3 g was observed in the control group at the end of the assay, while mice corresponding to the positive, carcinogen-treated group had a weight decrease after the administration of the agent (weeks three and four) with a subsequent recovery. Groups administered phaseolin had a mean weight increase of 4.7 g, and with respect to mice treated with both carcinogen and phaseolin, the response was apparently dose dependent, with increases of 3.3, 4.5, and 8.9 g with 40, 200, and 400 mg/kg phaseolin, respectively.

### 3.3. Antioxidant Effect

**Figure 2:**
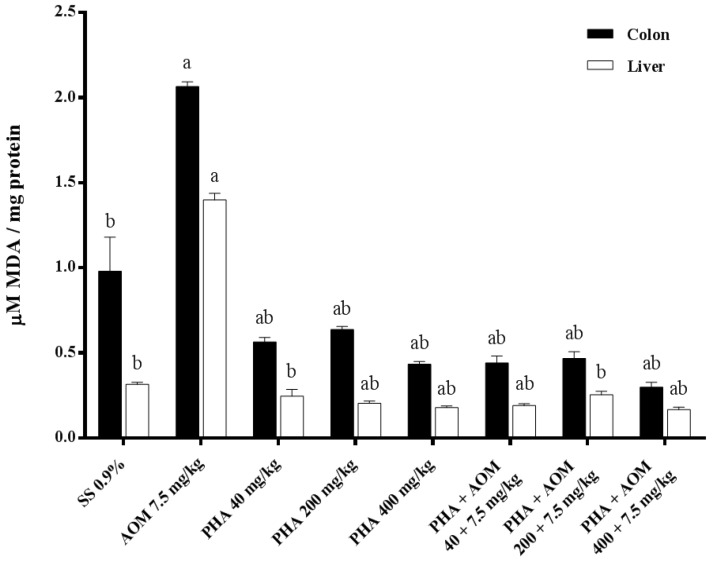
Effect of phaseolin (PHA) on the content of malondialdehyde (MDA) in the colon and liver of mice administered azoxymethane (AOM). Each bar represents the mean ± SEM obtained from six mice per group. ^a^ Statistically significant difference with respect to the saline solution (SS) control group, and ^b^ with respect to the AOM-treated group. ANOVA and Holm-Šídák tests, *p* < 0.05.

**Figure 3:**
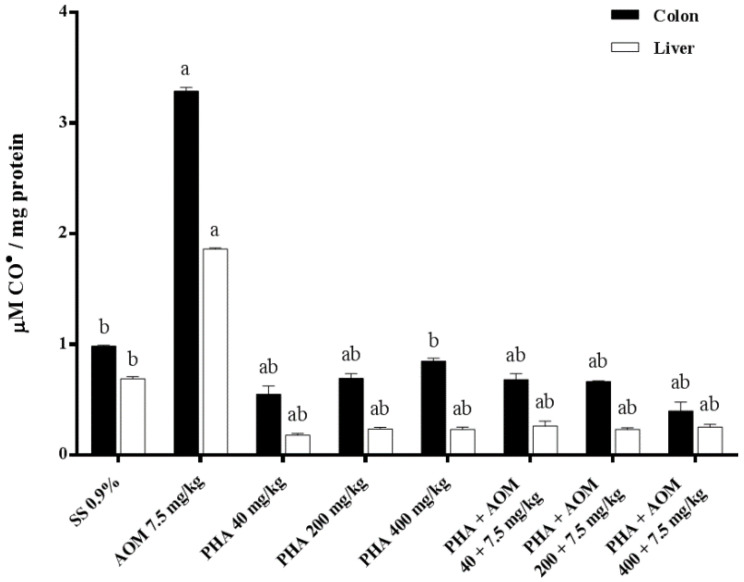
Effect of phaseolin on the carbonil (CO^•^) content in the colon and liver of mice administered azoxymethane (AOM). Each bar represents the mean ± SEM obtained from six mice per group. ^a^ Statistically significant difference with respect to the value obtained in the saline solution (SS) control group, and ^b^ with respect to the value obtained in the AOM-treated group. ANOVA and Holm-Šídák tests, *p* < 0.05.

The antioxidant capacity of phaseolin in the colon and liver is shown in Figure 2 and Figure 3. The first figure indicates the results obtained with respect to lipid peroxidation by measuring the level of malondialdehyde, and Figure 3 shows the results corresponding to protein oxidation through the measurement of reactive carbonyls. In the two examined organs, similar results were observed for both biomarkers. AOM produced significant oxidation in the colon and liver with respect to the control value; in the colon, the malondialdehyde increase was 112%, and in the liver, it was 348%, while the increase in oxidized proteins in the colon and liver was 224% and 173%, respectively. The three tested doses of phaseolin were unable to oxidize lipids or proteins by themselves; however, the previous administration of the three doses of bean protein to AOM-treated animals produced an oxidative reduction greater than 100% with respect to the value determined in AOM-treated mice.

**Figure 4:**
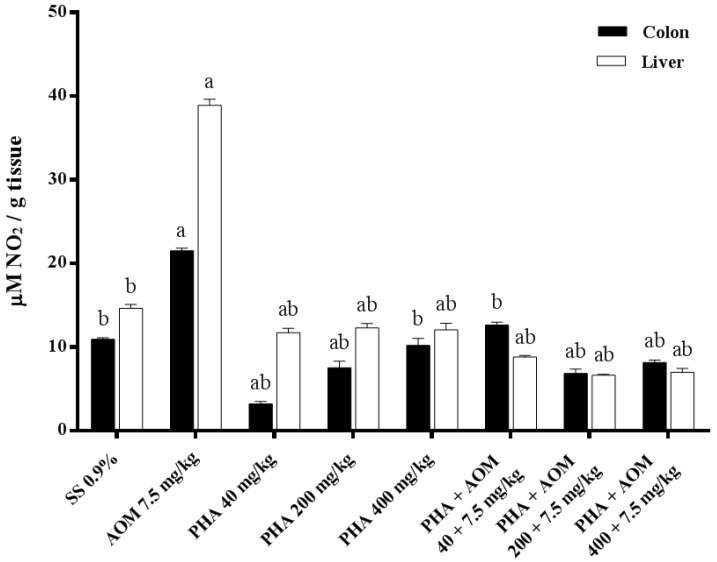
Effect of phaseolin (PHA) on the nitrite content (NO_2_) in the colon and liver of mice administered azoxymethane (AOM). Each bar represents the mean ± SEM obtained from six mice per group. ^a^ Statistically significant difference with respect to the saline solution (SS) control group, and ^b^ with respect to the value obtained in the AOM-treated group. ANOVA and Holm-Šídák tests, *p* < 0.05.

The antioxidant capacity of phaseolin was also determined by measuring the level of nitric oxide through the detection of nitrates/nitrites (Figure 4). Here, we observed an increase of more than twice the level of the studied parameter in both the colon and liver of mice treated with AOM in comparison with animals administered saline solution. A contrasting result was observed in mice administered phaseolin alone (40, 200, and 400 mg/mL), with the result found to be in the range of the control level. In addition, we confirmed that animals treated with phaseolin and later with AOM showed a strong reduction in nitrates/nitrites in comparison with AOM-treated mice, showing a level even better than that observed in the control group.

**Figure 5:**
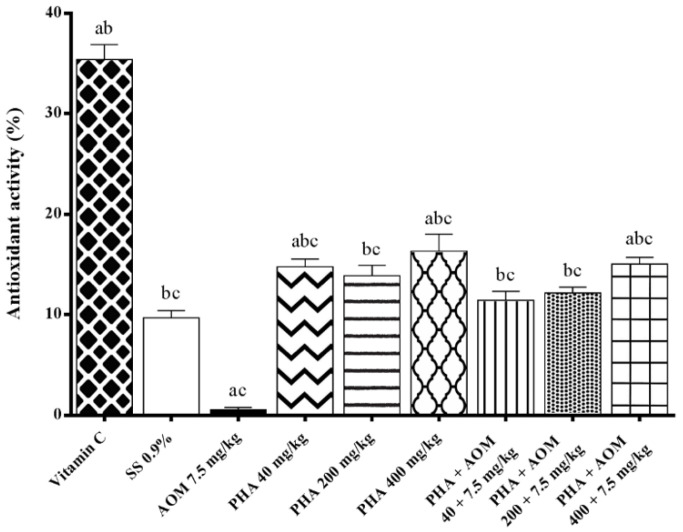
Antioxidant capacity of phaseolin (PHA) determined with the DPPH assay in the sera of mice treated with azoxymethane (AOM). Each bar represents the mean ± SEM from six mice per group (8 groups). ^a^ Statistically significant difference with respect to the control, saline solution (SS)-treated group, ^b^ with respect to the group treated with AOM, and ^c^ with respect to the value obtained in mice administered vitamin C. ANOVA and Holm-Šídák tests, *p* < 0.05.

Finally, we also examined the capacity of phaseolin to reduce DPPH radicals and found that the protein tested alone gave rise to a significant increase in DPPH radical capture in comparison with the results detected in AOM-treated animals and that the results obtained with the combination of phaseolin plus AOM, although slightly lower than the previous group, were also found to significantly increase the radical capture effect with respect to mice administered AOM only (Figure 5).

### 3.4. DNA Damage

**Figure 6:**
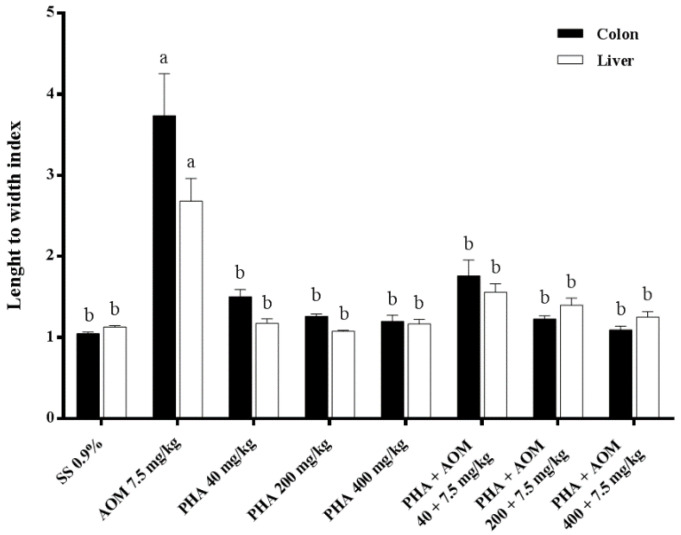
Effect of phaseolin (PHA) on the DNA damage induced with azoxymethane (AOM) in the colon and liver of mice. Each bar represents the mean ± SEM obtained from six mice per group, 100 nucleoids per mouse. ^a^ Statistically significant difference with respect to the control, saline solution (SS)-treated mice, and ^b^ with respect to the value obtained in the AOM-treated group. ANOVA and Holm-Šídák tests *p* < 0.05.

Figure 6 shows the results obtained with the comet assay in the colon and liver treated with AOM and phaseolin. With respect to the activity of AOM, we found a significant increase in the damage present in both organs in comparison with the control value. The DNA damage in the colon was 3.8 times higher than that observed in untreated mice, and in the liver, the increase was 2.7 times higher. The effect of the three doses of phaseolin showed a similar effect to that detected in the control animals, indicating that the examined protein had no DNA-damaging potential, while the combination of the three doses of phaseolin plus AOM gave rise to a significant preventive effect of the tested protein, yielding data very similar to those determined for the treatment with phaseolin alone.

### 3.5. Colon Aberrant Crypts

The total number of CAC and the multiplicity number are shown in Table 2. With respect to the first parameter, we found that the control and phaseolin-treated mice had a low number of aberrant crypts, with no more than a mean of 4.1 ± 1.1 crypts per colon, in contrast to mice administered AOM alone, which reached a mean of 100.4 ± 5.3 crypts per colon. In addition, animals treated with the combined compounds, that is, 40, 200, and 400 mg/kg phaseolin plus AOM administration, had a crypt mean number of 12.7, which represents a decrease of more than seven times with respect to the result obtained in AOM-treated animals. With regard to the multiplicity of crypts, we only found single crypts in both the control and phaseolin-treated animals. In the AOM-inoculated mice, however, we observed foci composed of up to seven crypts. Finally, in animals administered phaseolin and AOM, the observed foci were composed of no more than three crypts. The described results are in line with the phaseolin chemopreventive potential.

## 4. Discussion

In recent years, it has been demonstrated that beans and other legumes may exert a number of beneficial activities with respect to human health and that several of these actions are mainly related to non-nutritional compounds, such as polyphenols, saponins, protease or carbohydrase inhibitors. However, the structure and constitution of proteins and carbohydrates from legumes may also play an important role in the induced biomedical effects, which may be directly produced in the cell and the organism or as a consequence of their metabolism [47]. Therefore, in the present report, we selected phaseolin, a poorly studied bean protein, to evaluate its activity in three closely interconnected aspects: antioxidation, antigenotoxicity, and chemoprevention.

With respect to the first issue, our results showed that phaseolin completely abolished the lipid and protein oxidation induced by AOM in the colon and liver of mice, and therefore, it was concluded that the evaluated protein is a strong in vivo antioxidant; moreover, at the tested doses, the bean protein did not increase the oxidation of the studied biomolecules, a result that suggests no pro-oxidant capacity of the compound, as has been reported for other chemicals with antioxidant potential [48].

The importance of an oxidation-reduction equilibrium in the maintenance of the required homeostasis in the organism to correctly perform biological functions related to lipids, proteins and DNA, as well as to avoid or reduce the damage to health that may occur with an excessive amount of oxygen or nitrogen reactive species, is well documented [49]. As expected, in this area we found strong oxidation caused by AOM, a colon and liver carcinogen that has also been reported to induce damage in rat testes and lymphoid nodules. The chemical had previously been reported to be an oxidant based on its increase in ABTS radicals, peroxidase, glutathione, myeloperoxidase and malondialdehyde [50,51]; therefore, the oxidative inhibitory effect observed with phaseolin is an encouraging finding against the health damage related to the action of AOM but also based on the action of oxidative agents in general. In our present research, we also determined a similar response with respect to the DPPH radical: a drastic antioxidant decrease by AOM and an opposite, significant increase in the capture of the radical exerted by phaseolin in the AOM-treated animals that even reached the control range.

Our data in the antioxidant field agree with the in vitro results obtained by Carrasco-Castilla et al. [30] with purified or hydrolyzed phaseolin. In their study, the authors suggested that treatment with pepsin and pancreatin gave rise to the release of peptides with high antioxidant capacity that was shown with various methods: the beta-carotene bleaching assay, the reducing power, and the metal chelating activity. The authors also suggest the action of multiple mechanisms to explain their results, including the donation of hydrogen atoms and electrons, the stabilization of free radicals and the chelation of transition metals. Their observations clearly showed that phaseolin hydrolysis produced fragments with elevated antioxidant potential, an effect that we had previously observed in vivo concerning the hydrolyzed proteins of *Spirulina maxima* [52]; therefore, it is highly feasible that in our present assay, the in vivo metabolism could generate peptides and amino acids with elevated antioxidant capacity.

Single-cell gel electrophoresis (comet assay) is a versatile tool with good sensitivity, adaptability, and reliability that can be applied to most cells to measure DNA strand breaks, incomplete excision repair events, alkaline labile sites, and cross-linking events [53]. We applied this assay to determine the genotoxic effect induced by AOM, as well as the capacity of phaseolin to prevent such damage. Our results showed no DNA damage by the tested protein; in contrast, it was determined that the protein inhibited almost 100% of the deleterious effect induced by AOM in both examined organs. Various reports using beans have yielded congruent results. Lopes et al. [54] examined the genotoxic potential of white bean flour by means of the comet assay and found no effect, a result that was related to the presence of polyphenolic compounds, including tannins, terpenes, and lectins. In addition, Azevedo et al. [13] found no DNA damage with the comet and the micronucleus tests when cooked and dehydrated *P. vulgaris* (1–20%) were given to mice for 15 days; in contrast, they observed genoprotection against the damage induced by cyclophosphamide. The same authors also looked for compounds involved in the protective action and tested the effect of commercial anthocyanins, finding a DNA breaking effect with the high tested dose (50 mg/kg); therefore, they concluded that the protective effect of black bean may be due to the activity of various constituents. Our findings in the present study clearly suggest that one of these constituents may be phaseolin.

The mucosal surface of the colon contains openings to small invaginations known as crypts; however, in the development of colon cancer, these structures are modified to CAC and constitute the earliest morphologically observable lesions that have a direct association with the development of adenomas and adenocarcinomas [55]. Therefore, such lesions are very useful in examining the induction of pre-carcinogenesis and its prevention. Various authors have demonstrated a preventive effect of the non-digestible fraction or polysaccharide extracts of *P. vulgaris* against rodent AOM induction of CAC and have suggested modulation of genes involved in apoptosis, proliferation, cell cycle arrest, and inflammation as mechanisms of action [24,56,57]. With respect to chemoprevention, in the present report we demonstrated that the tested protein did not elevate the number of CAC; in contrast, the compound significantly reduced the number of such lesions in animals administered AOM. Our data showed that the preventive effect was exerted by the three tested doses of phaseolin and was higher than 80%.

Other legumes have also been studied with respect to their protective pre-carcinogenic or carcinogenic damage. Different forms of soybean have been evaluated and have shown controversial results; positive effects against the development of CAC or tumors have been reported, reaching a mean reduction of 56% in the frequency of crypts and 88% in the decrease in tumors [58,59,60,61,62,63]; however, negative results have also been obtained [64,65]. Regarding positive reports, Vuyyuri et al. [66] suggested the involvement of the bioactive peptide lunasin as a main participant because of its multiple functional domains that may modulate gene expression through different mechanisms. Chickpea is another studied legume. Its flour decreased the number of crypts by 64% in AOM-treated mice, probably related to the action of Bowman-Birk inhibitors and saponins [59]. Additionally, 2% and 10% cooked chickpea added to AOM/DSS-treated mice reduced the number of crypts by up to 95%, as well as various cell proliferation markers [67]. Finally, the same group showed that the hydrolysis of chickpea protein was able to reduce the number of aberrant crypts to the control level in mice administered AOM fed a hypercaloric diet [68].

Currently, it is common knowledge that genetics play a variable but important role in all disease processes, including common disorders, as a consequence of the multitude of differences in the organism’s DNA that may respond differently to the influence of environmental factors, including lifestyle, diet, exercise, or exposure to specific chemicals [69]. In this context, colon cancer is regarded as a multistep disease involving an initial mutagenic event called tumor initiation followed by additional mutagenic events and epigenetic activities known as tumor promotion, a process that culminates with the clinically detectable tumor that corresponds to the stage of cancer progression. A number of gene and chromosomal mutations as a consequence of genotoxic damage have been studied in this disease, including the *APC* gene, whose consequences may be highly increased with mutations in the *TP53* and *KRAS* genes [55]. Therefore, in this particular disease, the involvement of DNA damage is a relevant factor, and the detected prevention of such damage by phaseolin is also of interest because of its potential application. Within this line of research, cancer prevention, which started with the avoidance of deleterious lifestyle habits, later shifted to the identification of chemopreventive agents, as well as to the comprehension of their mechanism of action. At the present stage, the participation of oxidative processes in colon carcinogenesis is well known, as numerous agents with antioxidant properties have been shown to have preventive effects [70,71].

In our report, the preventive effect observed in the liver is also a significant finding in light of the worldwide relevance of diseases in this organ, which include hepatitis B virus and hepatitis C virus infections, alcoholic liver disease, non-alcoholic fatty liver disease and associated cirrhosis, liver failure, and hepatocellular carcinoma, accounting for approximately two million deaths per year worldwide: one million due to complications of cirrhosis and one million due to viral hepatitis and hepatocellular carcinoma [72]. In China in particular, liver diseases have been reported to affect approximately 300 million people [73].

Our present research concluded that phaseolin, a protein from *Phaseolus vulgaris*, is an agent with high antioxidant and antigenotoxic effects, as well as strong anti-carcinogenic potential, in the colon and liver of mice administered the carcinogen AOM.

## Figures and Tables

**Table 1:** Body weight determined in the experimental mice throughout the experiment.

Group (mg/kg)	Weight (g)							
	Week 0	Week 1	Week 2	Week 3	Week 4	Week 5	Week 6	Week 7
SS 0.9% (10 mL/kg)	25.5 ± 1.8	26.8 ± 2.6	27.2 ± 3.3 ^b^	27.7 ± 3.5 ^b^	28.3 ± 3.9 ^b^	29.4 ± 3.9	30.6 ± 4.6	31.8 ± 5.5
AOM (7.5)	24.8 ± 3.1	25.5 ± 3.0	22.3 ± 2.0 ^a^	21.1 ± 1.3 ^a^	22.5 ± 1.5 ^a^	24.1 ± 4.7	26.0 ± 2.4	27.5 ± 2.8
PHA (40)	26.6 ± 3.2	27.7 ± 4.2	28.1 ± 4.4 ^b^	28.4 ± 3.9 ^b^	28.6 ± 4.3 ^b^	29.5 ± 4.3	30.1 ± 4.4	31.0 ± 4.3
PHA (200)	24.7 ± 2.1	25.0 ± 2.4	25.3 ± 3.2	24.8 ± 3.5	26.3 ± 3.6	27.9 ± 3.2	28.6 ± 4.6	29.8 ± 4.2
PHA (400)	25.4 ± 3.3	26.2 ± 2.9	26.8 ± 2.5 ^b^	27.4 ± 3.8 ^b^	27.8 ± 3.5 ^b^	28.4 ± 3.6	29.3 ± 4.5	30.2 ± 5.2
PHA + AOM (40 + 7.5)	26.8 ± 2.8	26.7 ± 2.8	24.4 ± 2.0	24.1 ± 3.1	25.4 ± 2.7	27.3 ± 3.5	28.6 ± 3.8	30.1 ± 3.3
PHA + AOM (200 + 7.5)	25.5 ± 2.7	26.9 ± 3.8	25.1 ± 3.3	24.3 ± 3.1	24.8 ± 2.4	26.5 ± 3.1	28.1 ± 3.8	30.0 ± 3.9
PHA + AOM (400 + 7.5)	24.6 ± 1.2	26.5 ± 1.0	25.8 ± 1.7	26.9 ± 3.3 ^b^	28.8 ± 2.6 ^b^	30.6 ± 3.3 ^b^	32.0 ± 2.2	33.5 ± 3.2

PHA = phaseolin. Each value corresponds to the mean ± SEM obtained from three independent measurements per week in 12 mice per group. ^a^ Statistically significant difference with respect to the saline (SS) control group, and ^b^ with respect to the value obtained in the azoxymethane (AOM)-treated group. ANOVA and Holm-Šídák, *p* < 0.05.

**Table 2:** Effect of phaseolin (PHA) on the number and multiplicity of colon aberrant crypts (CAC) in the colon of mice.

Treatment (mg/kg)	Number of Colon Aberrant Crypts (CAC)/Focus							CAC/Colon (Number)	Focus/Colon (Number)
	1	2	3	4	5	6	7		
SS 1% (10 mL/kg)	4.1 ± 1.1	-	-	-	-	-	-	4.1 ± 2.7 ^b^	4.1 ± 2.7 ^b^
AOM (7.5)	37 ± 3.5	11.8 ± 1.2	5.6 ± 1.4	2.6 ± 0.3	2 ± 0.4	0.2 ± 0.1	0.2 ± 0.1	100 ± 10.8 ^a^	59.5 ± 7.5 ^a^
PHA (40)	2.8 ± 0.6	-	-	-	-	-	-	2.8 ± 1.4 ^b^	2.8 ± 1.4 ^b^
PHA (200)	3 ± 0.7	-	-	-	-	-	-	3 ± 1.7 ^b^	3 ± 1.7 ^b^
PHA (400)	3.1 ± 0.9	-	-	-	-	-	-	3.1 ± 2.3 ^b^	3.1 ± 2.3 ^b^
PHA + AOM (40 + 7.5)	7.8 ± 2.1	2.3 ± 0.6	0.8 ± 0.4	-	-	-	-	15 ± 7.0 ^ab^	11 ± 5.5 ^b^
PHA + AOM (200 + 7.5)	9.5 ± 1.3	1.6 ± 0.6	1 ± 0.6	-	-	-	-	15.8 ± 8.7 ^ab^	12.1 ± 4.7 ^ab^
PHA + AOM (400 + 7.5)	13.3 ± 1.5	1.3 ± 0.5	0.5 ± 0.3	-	-	-	-	17.5 ± 6.8 ^ab^	15.1 ± 4.7 ^ab^

Each value represents the CAC mean number ± SEM obtained in the whole colon of six mice per group. ^a^ Statistically significant difference with respect to saline solution (SS) and ^b^ with respect to the value obtained in the azoxymethane (AOM)-treated group. ANOVA and Holm-Šídák tests, *p* < 0.05.

## Data Availability

Data supporting results are in the Genetics Laboratory, National School of Biological Sciences, National Polytechnic Institute, México.
